# Supplementary material for: Expression plasticity regulates intraspecific variation in the acclimatization potential of a reef-building coral
Source: Nat Commun. 2022 Aug 15;13:4790. doi: 10.1038/s41467-022-32452-4 (PMC9378650; doi:10.1038/s41467-022-32452-4)
Supplement: Supplementary file 1 — Supplementary Information [file 41467_2022_32452_MOESM1_ESM.pdf]

Supplementary Information for:

Expression plasticity regulates intraspecific variation in the acclimatization potential of a reef-building coral

Crawford Drury[1*], Jenna Dilworth[1,2], Eva Majerová[1], Carlo Caruso[1], Justin B Greer[3]

[1] Hawaiʻi Institute of Marine Biology, Kāneʻohe, HI
[2] University of Southern California, Los Angeles, CA
[3] U.S. Geological Survey, Western Fisheries Research Center, Seattle, WA

*Correspondence: crawford.drury@gmail.com

Supplemental Table 1 - Gene ontologies distinguishing Constant High Treatment

Gene ontologies in this table were significant (FDR P<0.1) enriched in high z-scores, which distinguish the constant high treatment from all others (pulse, pulse increase, pulse high).

Name	GO Term	Number	P (FDR)	Class
peptide biosynthetic process	GO:0006412;GO:0043043	100	0.033	BP
peptide metabolic process	GO:0006518	120	0.017	BP
lipid metabolic process	GO:0006629	106	0.049	BP
movement of cell or subcellular component	GO:0006928	133	0.031	BP
cell migration	GO:0016477	73	0.049	BP
cellular amide metabolic process	GO:0043603	142	0.049	BP
locomotion	GO:0048870;GO:0040011	89	0.049	BP
cellular response to endogenous stimulus	GO:0071495	44	0.049	BP
response to oxygen-containing compound	GO:1901700	95	0.049	BP
structural constituent of ribosome	GO:0003735	85	0.011	MF
structural molecule activity	GO:0005198	143	0.003	MF

Supplemental Table 2 - **Significant HMMER matches for Cluster 77005**

Cluster 77005 was identified as NCBI Gene ID 107331382, an uncharacterized locus in *A. digitifera*. Orthologs identified with OrthoDB indicated AAA-type ATPases. Further functional annotation of the predicted protein sequence, XP_015751448.1, was investigated using hidden Markov Models in HMMER. Each of the most significant hits contained an AAA+ ATPase domain (InterPro domain IPR003593). Several predicted nephrocystin-3 transcripts in *S. pistillata* were indicated, and only the lowest e-value match of these is presented.

UniProt ID	Gene Name	Species	e-value
A0A2B4R855	Nephrocystin-3	*Stylophora pistillata*	6.43e-105
A0A3M6UWQ2	ATPase_AAA_core domain-containing protein	*Pocillopora damicornis*	2.5e-68
A0AB4R9I8	Putative WD repeat-containing protein alr2800	*Stylophora pistillata*	7.8e-61

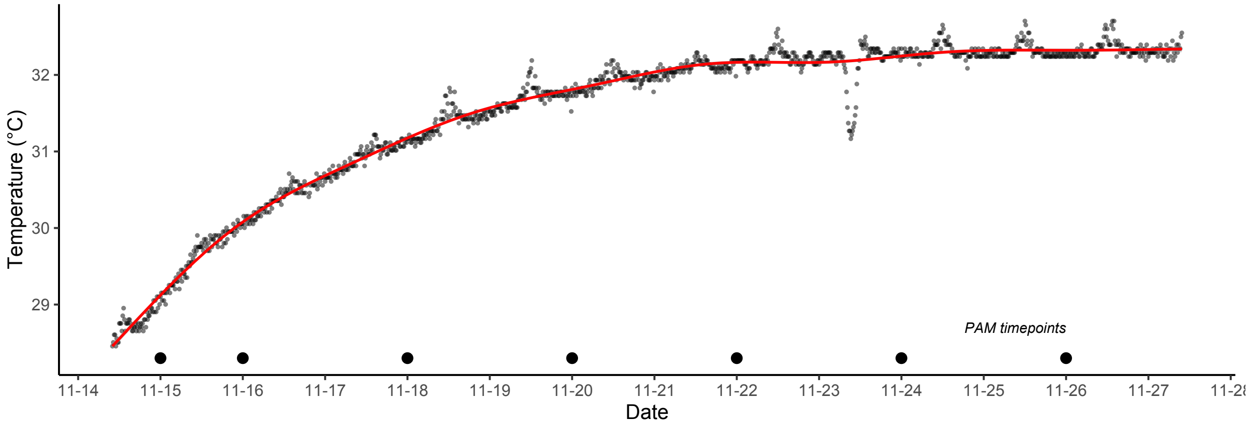

Supplemental Figure 1 - Stress-testing Profiles

Timeline of experimental stress-testing profiles occuring 124 days after pre-exposure. Line is loess smoothing of temperaure and black dots at the bottom represent PAM timepoints used for dose response curve fitting.

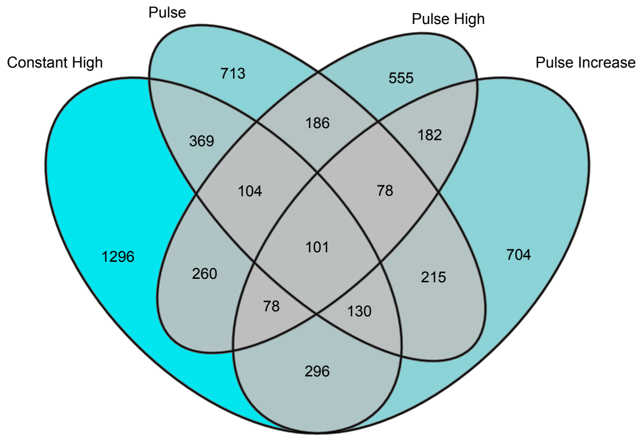

Supplemental Figure 2 - Shared Differentially Expressed Genes Between Treatmetns

Venn diagram of differnetially expressed genes comparing each pre-exposure treatment to control ($p<0.01$). Color represents shading of individual segments based on number of differentially expressed genes.
