# Supplementary material for: Expression plasticity regulates intraspecific variation in the acclimatization potential of a reef-building coral
Source: Nat Commun. 2022 Aug 15;13:4790. doi: 10.1038/s41467-022-32452-4 (PMC9378650; doi:10.1038/s41467-022-32452-4)
Supplement: Supplementary file 3 — Reporting Summary [file 41467_2022_32452_MOESM3_ESM.pdf]

nature portfolio

Corresponding author(s): Crawford Drury

Last updated by author(s): July 21, 2022

Reporting Summary

Nature Portfolio wishes to improve the reproducibility of the work that we publish. This form provides structure for consistency and transparency in reporting. For further information on Nature Portfolio policies, see our Editorial Policies and the Editorial Policy Checklist.

Statistics

For all statistical analyses, confirm that the following items are present in the figure legend, table legend, main text, or Methods section.

n/a	Confirmed	
[ ]	[x]	The exact sample size (*n*) for each experimental group/condition, given as a discrete number and unit of measurement
[ ]	[x]	A statement on whether measurements were taken from distinct samples or whether the same sample was measured repeatedly
[ ]	[x]	The statistical test(s) used AND whether they are one- or two-sided<br>*Only common tests should be described solely by name; describe more complex techniques in the Methods section.*
[ ]	[x]	A description of all covariates tested
[ ]	[x]	A description of any assumptions or corrections, such as tests of normality and adjustment for multiple comparisons
[ ]	[x]	A full description of the statistical parameters including central tendency (e.g. means) or other basic estimates (e.g. regression coefficient) AND variation (e.g. standard deviation) or associated estimates of uncertainty (e.g. confidence intervals)
[ ]	[x]	For null hypothesis testing, the test statistic (e.g. *F*, *t*, *r*) with confidence intervals, effect sizes, degrees of freedom and *P* value noted<br>*Give P values as exact values whenever suitable.*
[x]	[ ]	For Bayesian analysis, information on the choice of priors and Markov chain Monte Carlo settings
[x]	[ ]	For hierarchical and complex designs, identification of the appropriate level for tests and full reporting of outcomes
[ ]	[x]	Estimates of effect sizes (e.g. Cohen's *d*, Pearson's *r*), indicating how they were calculated

Our web collection on statistics for biologists contains articles on many of the points above.

Software and code

Policy information about availability of computer code

Data collection	Walz WinControl-3 was used to record PAM flouremetry data.
Data analysis	We used salmon 1.14.0 to align and quanify data, cutadapt 3.7 to trim raw reads and custom code (tagseq_clipper.pl) to remove duplicate and PCR reads, available at https://github.com/z0on/tag-based_RNAseq. We used bwa-mem 0.7.17 to align metatranscriptome data to the reference for alignment preparation and cd-hit est 4.8.1 to find consensus sequences. We used blastx 2.12.0 to annotate contigs of interest. We used MMotifFinder (https://www.genome.jp/tools/motif/), OrthoDB (orthodb.org), Interpro (ebi.ac.uk/Interpro), Pfam (pfam.xfam.org) to annotate data.<br><br>The following software was used to analyze data:<br>R 4.1.2, GO_MWU (https://github.com/z0on/GO_MWU), vegan 2.5, drc 3.0, deseq2 1.34

For manuscripts utilizing custom algorithms or software that are central to the research but not yet described in published literature, software must be made available to editors and reviewers. We strongly encourage code deposition in a community repository (e.g. GitHub). See the Nature Portfolio guidelines for submitting code & software for further information.

Data

Policy information about availability of data

All manuscripts must include a data availability statement. This statement should provide the following information, where applicable:

- Accession codes, unique identifiers, or web links for publicly available datasets
- A description of any restrictions on data availability
- For clinical datasets or third party data, please ensure that the statement adheres to our policy

Raw sequencing data is available at NCBI under Bioproject PRJNA847955. The pfam database is hosted at pfam.xfam.org, the InterPro database is hosted at ebi.ac.uk/Interpro, the OrthoDB database is hosted at orthodb.org.

Field-specific reporting

Please select the one below that is the best fit for your research. If you are not sure, read the appropriate sections before making your selection.

☐ Life sciences ☐ Behavioural & social sciences ☒ Ecological, evolutionary & environmental sciences

For a reference copy of the document with all sections, see nature.com/documents/nr-reporting-summary-flat.pdf

Ecological, evolutionary & environmental sciences study design

All studies must disclose on these points even when the disclosure is negative.

Study description	Fifty replicates of each of ten genotypes of Montipora were collected, returned to HIMB, mounted and acclimated to indoor seawater tanks. Fragments were evenly divided into ten tanks and exposed to one of five five-day temperature treatments including a control) in replicate tanks, with RNA samples collected before and after exposure. 300 fragments (n=5-7/genotype/treatment) were returned to the collection site for ~3 months before being stress tested in replicate tanks.<br><br>Initial timepoint: N=5 RNA samples/genotype equating to 25 RNA samples/phenotype. Final timepoint: N=5 RNA samples/phenotype/treatment.
Research sample	Ten genotypes of Montipora capitata sourced from a single reef (13) in Kaneohe Bay with known historical bleaching phenotypes. The dataset was chosen to maximize availble experimental context from previous work and is meant to represent this species from Kaneohe Bay.
Sampling strategy	Sample size was determined arbitrarily based on availability of coral material adn tank space, with a focus on maximizing replication to increase power in the downstream stress testing.
Data collection	Data was collected by Drury, Dilworht and Caruso. RNA samples were haphazardly assigned to differnet collectors within each timepoint. PAM data was collected blind to treatment, genotype and phenotype.
Timing and spatial scale	Corals were collceted from a single reef, all colonies were located within a ~100m linear area.<br><br>Corals were collected in June 2019, treated for five days from July 9-13 2019, returend to the reef on august 15th and recollected for stress testing on November 20 2019. Stress testing was evaluated via PAM flourometry every other day for the duraiton of the experiment.
Data exclusions	No data were excluded.
Reproducibility	Due to logistics and the long duration of this experiment, we did not attempt to reproduce our results. Biologically relevant reproducbiilty (i.e., recapitualtion of expected bleaching patterns relative to phenotype) was observed.
Randomization	Corals were haphazardly selected from a larger library of individuals and fragmented. Coral fragments were randomly allocated into tanks in equal numbers across replicates and treatments.
Blinding	PAM data was collected on fragments on large racks identified only by numeric plug numbers, which blinds the observers to treatment, phenotype and genotype.

Did the study involve field work? ☒ Yes ☐ No

Field work, collection and transport

Field conditions	Field collections were made at a single shallow reef in Kaneohe Bay during June 2019.
Location	Study materials were collected from Reef 13 in Kaneohe Bay within 50m of 21.4511356, -157.796417. All colonies in the experiment were at 2-4m depth

Access & import/export	The site was accessed by boat and did not interfere with the environment. Adult and gamete collections were made under Hawaii Department of Land and Natural Resources permit SAP 2020-25 issued to the Hawaii Institute of Marine Biology in June 2019.
Disturbance	Aside from sampling permitted corals, there was no disturbance to the ecosystem.

Reporting for specific materials, systems and methods

We require information from authors about some types of materials, experimental systems and methods used in many studies. Here, indicate whether each material, system or method listed is relevant to your study. If you are not sure if a list item applies to your research, read the appropriate section before selecting a response.

Materials & experimental systems

n/a	Involved in the study	
☒	☐	Antibodies
☒	☐	Eukaryotic cell lines
☒	☐	Palaeontology and archaeology
☐	☒	Animals and other organisms
☒	☐	Human research participants
☒	☐	Clinical data
☒	☐	Dual use research of concern

Methods

n/a	Involved in the study	
☒	☐	ChIP-seq
☒	☐	Flow cytometry
☒	☐	MRI-based neuroimaging

Animals and other organisms

Policy information about studies involving animals; ARRIVE guidelines recommended for reporting animal research

Laboratory animals	This study did not involve laboratory animals.
Wild animals	Corals were collected using bone clippers in situ, placed in a cooler of ambient seawater and returned to the Hawaii Institute of Marine Biology within ~30 minutes by boat. We collected 50 ~5cm fragments from each of 10 large adult Montipora capitata colonies, which is a hermaphrodite. Samples experienced mortality during stress testing.
Field-collected samples	Ten known genotypes of Montipora capitata [41] were collected from Reef 13 in Kāneʻohe Bay in May 2019, equally representing each of two historical bleaching phenotypes originally tagged in the 2015 bleaching event [42, 43]. These samples were equally split between Cladocopium- and Durusdinium-dominated colonies [44]. Fragments (n=500) were returned to the Hawaiʻi Institute of Marine Biology, mounted on labeled plugs, and transferred to indoor mesocosms (Figure 1) with ambient sand-filtered seawater (~27.5C) and a 12h:12h light:dark cycle at a maximum irradiance of ~383 umol sec-1. Mesocosms were cleaend as needed. After two weeks of indoor acclimation, corals were exposed to either a control or one of four short-term heat pre-exposure profiles (constant high, pulse, pulse increase, pulse high) between 28°C and 31.5°C for 5 days (Figure 1b) in replicate tanks (n=2). After this pre-exposure, a subset of corals (n=5-7 fragments per colony per treatment, N=299) were returned to the collection reef on August 15th and mounted on a platform at ~2m depth for recovery prior to subsequent stress testing. Samples experienced mortality at the end of the experiment during stress testing.
Ethics oversight	Aside from colelction permits, the state of Hawaii does not require oversight of invertebrates. . Corals were collected under DAR permit# SAP-2020-25 to HIMB.

Note that full information on the approval of the study protocol must also be provided in the manuscript.
